# Successful treatment of anti-LGI1-associated autoimmune encephalitis with efgartigimod: A case report and literature review

**DOI:** 10.1097/MD.0000000000048535

**Published:** 2026-05-15

**Authors:** Rui Gao, Yan Hong, Guanen Zhou

**Affiliations:** a Department of Neurology, Tianjin Key Laboratory of Cerebral Vascular and Neurodegenerative Diseases, Tianjin Huanhu Hospital, Tianjin, China.

**Keywords:** autoimmune encephalitis, cognitive impairment, efgartigimod, immunotherapy, LGI1 antibody

## Abstract

**Rationale:**

Anti leucine rich glioma inactivated 1 (LGI1)- associated autoimmune encephalitis (AE) is the common form of AE, The treatments for AE may cause many adverse effects and some treatments rely on limited blood resources. Monoclonal antibodies directed against neonatal crystallizable fragment receptor (FcRn), efgartigimod, accelerate autoantibody degradation by blocking IgG recirculation. The efficacy of efgartigimod has been demonstrated in myasthenia gravis. However, there are few case reports on the treatment of anti-LGI1-associated AE with efgartigimod. In this study, we present the case of a patient with anti-LGI1-associated AE who was treated with efgartigimod successfully.

**Patient concerns:**

A 64-year-old female patient presented with decline in short-term memory and limb tremor. Cranial magnetic resonance imaging (MRI) scan showed hyperintensity and swelling in the medial aspect of the right temporal-occipital lobe on T2- and FLAIR-weighted sequences, without obvious enhancement lesions. The anti-LGI1 antibody titer in serum and CSF was positive.

**Diagnoses:**

The patient was diagnosed with anti-LGI1-associated AE.

**Interventions:**

Initially, the patient received methylprednisolone pulse therapy. Levetiracetam and oxcarbazepine were both administered orally in the meantime. Following treatment failure, efgartigimod was administered once a week for 3 weeks.

**Outcomes:**

After treatment, the patient’s limb tremors ceased and her memory improved. The level of anti-LGI1 antibody titer in serum decreased, and the abnormal signals on the MRI attenuated.

**Lessons:**

Efgartigimod is an effective treatment for anti-LGI1-associated AE. This study presents a new approach to treat anti-LGI1-associated AE. However, further research is needed to establish the optimal dosage and frequency of efgartigimod, and to clarify its long-term safety and efficacy.

## 1. Introduction

AE is an autoimmune disease caused by autoantibodies that target neuronal or synaptic proteins. Anti-LGI1-associated AE is an important subtype, accounting for approximately 20% of cases.^[1,2]^ Anti-LGI1-associated AE has an acute or subacute onset. It mostly affects middle-aged and elderly individuals and is more prevalent in males.^[3]^ The clinical features include memory disturbance, neuropsychiatric symptoms, facial-brachial dystonic seizures (FBDS) and hyponatremia.^[4,5]^ The primary treatment for anti-LGI1-associated AE is immunotherapy. The first-line treatment includes steroids, intravenous polyclonal immunoglobulins(IVIg), and plasma exchange(PE). If the first-line treatments are ineffective, second-line treatments such as rituximab will be considered.^[6]^ However, there are potential risks associated with IVIg and PE, and they rely on limited blood resources. In addition, some patients experience poor outcomes or relapse. Therefore, the necessity to explore innovative targeted therapies is paramount.

In recent years, novel drugs that target B cells and antibody clearance have gradually become more widely used in the treatment of AE. The FcRn controls the trafficking and recycling of the pathogenic IgG, playing a critical role in protecting it from degradation by intracellular lysosomes and contributing to its extended half-life.^[7]^ Efgartigimod, a monoclonal antibody that targets FcRn, accelerates the degradation of pathogenic autoantibodies by blocking the recirculation of IgG. However, it does not significantly affect other immunoglobulin subclasses, such as immunoglobulin A and immunoglobulin M.^[8,9]^ Efgartigimod has been approved for the treatment of myasthenia gravis.^[8,10]^ Its effectiveness in treating other IgG antibody-mediated diseases such as Guillain-Barré syndrome and neuromyelitis optica spectrum disorder has also been demonstrated.^[11,12]^ Nevertheless, there is a paucity of case reports concerning the treatment of anti-LGI1-associated AE with efgartigimod. In this study, we present a case of anti-LGI1-associated AE successfully treated with methylprednisolone combination with efgartigimod. Efgartigimod significantly improve the symptoms of patients without any obvious side effects. Our report confirms the efficacy and safety of efgartigimod for treating anti-LGI1-associated AE. This provides a reference for optimizing the treatment strategy for anti-LGI1-associated AE.

## 2. Case presentation

**Figure 1.**
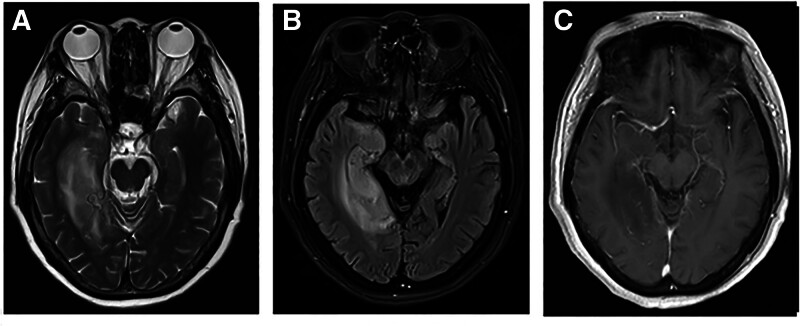
Baseline cranial MRI scan features of the patient before treatment. (A) The abnormal hyperintensity and swelling in the medial aspect of the right temporal-occipital lobe on T2-weighted sequence. (B) The abnormal hyperintensity and swelling in the medial aspect of the right temporal-occipital lobe on FLAIR-weighted sequence. (C) The lesions in the medial aspect of the right temporal-occipital lobe exhibit no contrast enhancement.

A 64-year-old female patient experienced a significant decline in short-term memory with dizziness for 10 days, accompanied by occasionally limb tremor (most prominent in the left lower limb), with no disturbance of consciousness. There was no fever, headache or abnormal behavior. The patient had a previous history of hypertension and depression. The physical examination of the nervous system revealed impaired calculation ability, memory loss, and an involuntary tremor in the left limb. The Mini-Mental State Examination (MMSE) was performed on the day of hospitalization, with a score of 24. The laboratory tests showed serum sodium 134 (mmol/L). There were no significant abnormalities in routine blood tests, liver and kidney function tests, or tumor marker screening. Baseline cranial MRI scan showed hyperintensity and swelling in the medial aspect of the right temporal-occipital lobe on T2- and FLAIR-weighted sequences, with no contrast enhancement (Fig. 1). Magnetic resonance spectroscopy (MRS) Showed an abnormal signal in the medial aspect of the right temporal-occipital region. Cho was not significantly increased, NAA was decreased, and a Lac peak was observed. No obvious proliferative changes were evident. Electroencephalogram examinations showed a slight increase of the intermittent slow waves in the anterior head, without any definite epileptic waves. The lumbar puncture was performed and the pressure was normal. The cerebrospinal fluid (CSF) examination showed a slight increase in glucose (5.07 mmol/L), while its appearance, white blood cell count, protein and chloride levels, and immunoglobulin levels were normal. The anti-LGI1 antibody titers in serum and CSF were 1:32 and 1:3.2, respectively. Paraneoplastic syndrome antibody profiles and pathogenic microorganism sequencing were negative. The patient was diagnosed with anti-LGI1-associated AE.

**Figure 2.**
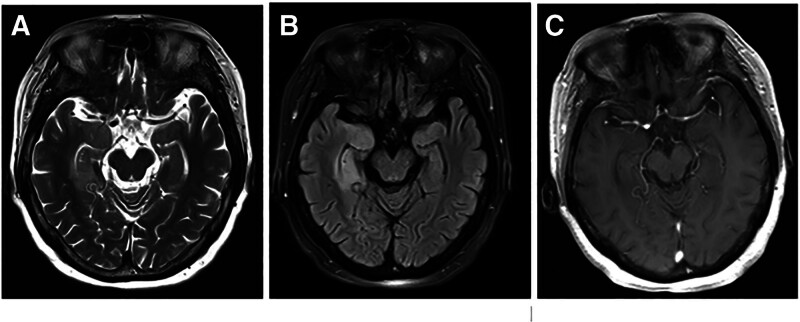
Repeat cranial MRI scan features of the patient after 3 weeks of efgartigimod treatment. (A) The abnormal signals in the medial aspect of the right temporal-occipital lobe on T2 -weighted sequence were attenuated compared to the baseline cranial MRI scan. (B) The abnormal signals in the medial aspect of the right temporal-occipital lobe on FLAIR-weighted sequence were attenuated compared to the baseline cranial MRI scan. (C) The lesions in the medial aspect of the right temporal-occipital lobe exhibit no contrast enhancement.

**Figure 3.**
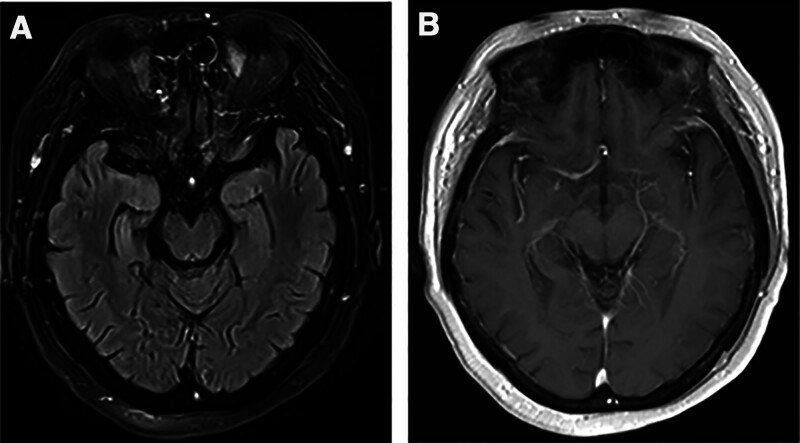
Repeat cranial MRI scan features of the patient after 3 months of efgartigimod treatment. (A) The abnormal signals in the medial aspect of the right temporal-occipital lobe on FLAIR-weighted sequence were further attenuated compared to the second cranial MRI scan. (B) The lesions in the medial aspect of the right temporal-occipital lobe exhibit no contrast enhancement.

**Figure 4.**
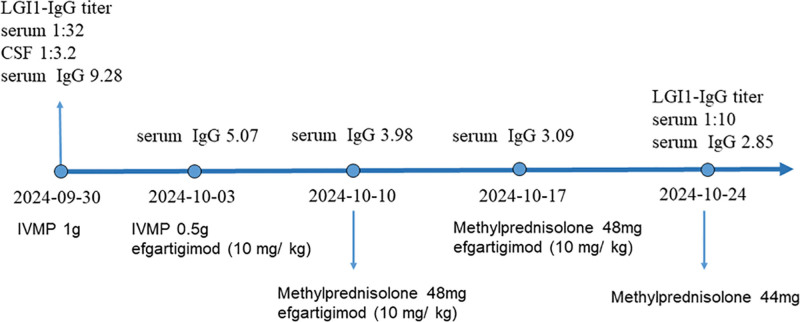
Timeline of the laboratory findings and treatments. Following methylprednisolone pulse therapy combined with 3 cycles of efgartigimod, the serum LGI antibody titers and IgG levels both decreased. LGI1 = leucine rich glioma inactivated 1, IgG = immunoglobulin G, CSF = cerebrospinal fluid, IVMP = intravenous methylprednisolone.

The patient was treated with intravenous methylprednisolone (1 g) for 3 days. Meanwhile levetiracetam (0.5 g) and oxcarbazepine (0.15g) were both administered orally twice a day. After treatment, there was no significant improvement in the patient’s symptoms, and the limb tremors continued to recur. With the family’s written consent, we administered intravenous efgartigimod (10 mg/kg) once a week to further improve symptoms. At the same time, gradually reduce the methylprednisolone dose as follows: 500 mg IV on days 4 to 6, then 48 mg orally on days 7 to 20, and subsequently reduce by 4 mg every 2 weeks. After 3 weeks of treatment, the level of IgG in the serum decreased to 2.85 (g/L), therefore, we discontinued the application of efgartigimod. The level of anti-LGI1 antibody in serum decreased to 1:10. The score of MMSE test was 28. The patient experienced no dizziness or limb tremor, and their calculation and memory abilities improved. The patient underwent repeat cranial MRI scans after 3 weeks (Fig. 2) and 3 months (Fig. 3) of efgartigimod treatment. The results showed that the lesions in the right temporo-occipital lobe gradually attenuated and with no contrast enhancement. No adverse events were observed during the 3-month follow-up of period. Figure 4 shows a timeline of the laboratory findings and treatments.

## 3. Literature review

Here, we provided a summary of the clinical characteristics of efgartigimod in the treatment for autoimmune encephalitis (Table 1).^[13–16]^ We conducted a systematic literature review to provide an overview of the demographic characteristics, dosing frequency, antibody titers and prognosis of autoimmune encephalitis treated with efgartigimod. On July 19, 2025, we searched the medical database PubMed using the terms “autoimmune encephalitis” and “efgartigimod” and additionally checked the respective reference lists. Previous studies reported 6 cases of autoimmune encephalitis treated with efgartigimod, including 5 male patients and 1 female patient, ranging in age from 4 to 67 years old. Anti-NMDAR and anti-LGI1 associated autoimmune encephalitis were reported more frequently, including 1 case of dual antibody (anti- NMDAR and anti-LGI1). The basic treatment for anti-LGI1-associated AE was cortisol, and for anti-NMDAR- associated AE or AE with coexistent NMDAR and LGI1 antibodies were cortisol combined with IVIG. The dosing frequency of efgartigimod varies from 2 to 4 cycles. After treatment with efgartigimod, the antibody titers in serum and cerebrospinal fluid decreased. All patients showed improvement in clinical symptoms and none experienced adverse effects. It is worth noting that in a case reported by Huang et al,^[14]^ the patient’s symptoms improved after 2 cycles of efgartigimod treatment. The treatment was then discontinued. However, the symptoms recurred 2 weeks later. After 2 more cycles of efgartigimod treatment, the symptoms gradually relieved. The patient was followed up for 2 months and there was no recurrence.

**Table 1:** Summary of the demographic characteristics, dosing frequency, antibody titer and prognosis of autoimmune encephalitis treated with efgartigimod in the literature.

Authors	Zhu F et al^[13]^	Huang H^[14]^	Zhang Q^[15]^	Zhang Q^[15]^	Zhang Q^[15]^	Xu J^[16]^
Years	2024	2025	2024	2024	2024	2025
Age	50	42	60	38	68	32
Gender	male	male	male	male	female	male
AE subtype	LGI1	NMDAR	GABABR	LGI1	NMDAR	NMDAR LGI1
Basic treatment	Cortisol	IVIG and cortisol	IVIG and cortisol	Cortisol	IVIG and cortisol	IVIG and cortisol
Dosing frequency	2 cycles	2 cycles first, Discontinued for 2 weeks, Reapply 2 cycles	4 cycles	4 cycles	4 cycles	3 cycles
Antibody titer (pretreatment)	1:100 (serum) 1:3.2 (CSF)	1:100 (serum) 1:100 (CSF)	1:100 (serum) 1:10 (CSF)	1:100 (serum)	1:10 (serum)	NMDAR 1:100 (serum) 1:32 (CSF) LGI1 1:10 (serum) 1:1 (CSF)
Antibody titer (after efgartigimod treatment)	1:10 (serum)	1:10 (serum) 1:1 (serum)	1:32 (serum)	1:10 (serum)	Refusal to detect	NMDAR 1:32 (serum) 1:10 (CSF) LGI1 1:10 (serum) negative in CSF
Recurrence	No	Yes	No	No	No	No
Adverse event	No	No	No	No	No	No

CSF = cerebrospinal fluid, GABABR = g-aminobutyric acid-B receptor, IVIG = intravenous polyclonal immunoglobulins, LGI1 = leucine rich glioma inactivated 1, NMDAR = N-methyl-D-aspartate receptor.

## 4. Discussion

This patient with anti-LGI1-associated AE was treated with methylprednisolone pulse therapy initially, but this was ineffective. After treatment with efgartigimod, the patient showed significant improvement in clinical symptoms and imaging abnormalities, and the level of anti-LGI1 antibodies in serum decreased. At the same time, no adverse events such as infections or headaches occurred, and there were no relapses. This study demonstrated that efgartigimod, a new immunotherapy drug, has shown excellent efficacy in the treatment of anti-LGI1-associated AE.

LGI1 is a secreted glycoprotein mainly expressed in the brain,^[17]^ which plays an important role in the development of the nervous system. Studies show that LGI1 promotes the growth of neurites and the formation and maturation of synapses. In addition, it has been shown to regulate the formation of neuronal myelin sheaths and the function of neuronal ion channels.^[18,19]^ Loss of LGI1 function due to autoantibodies results in the anti-LGI1-associated AE.^[5,20]^ The motor cortex and mesial temporal lobe of the brain are primarily affected by anti-LGI1-associated AE, leading to the different clinical phenotypes such as FBDS and limbic encephalitis.^[21]^ Scientists tend to prefer the term tonic dystonic seizure (TDS) to FBDS because the involvement of the face can be variable or absent.^[22]^ Reports have shown that FBDS precede limbic encephalitis in approximately 75% of cases.^[23]^ As patients progress clinically, they present with established LE and increasingly abnormal investigation findings, encompassing temporal lobes hyperintensities on T2-weighted sequences, ictal electroencephalographic abnormalities, and hyponatremia.^[4,24]^ TDS is often refractory to antiepileptic drugs but responds well to immunosuppressive and immunomodulatory therapies.^[25]^ Studies suggest that early immunotherapy may impede the progression from FBDS to LE.^[26]^ The patient in our study presented with atypical facial-brachial dystonic seizures, memory loss, the anti-LGI1 antibody positivity and temporal lobe hyperintensities on T2- and FLAIR-weighted sequences. This case is definitively diagnosed with anti-LGI1-associated AE.

The immune mechanisms underlying the production of pathogenic antibodies have been well demonstrated. B cells are a critical component of the adaptive immune system, and some eventually differentiate into plasmablasts or plasma cells, which are responsible for producing all antibodies in vivo. In the healthy state, antibodies are a defense against infection. There are immune tolerance mechanisms in the healthy state, which refers to the limitation of self-reactivity by functional immune checkpoints.^[27]^ However, in autoantibody-mediated diseases, autoreactive B cells evade tolerance checkpoints and decrease the tolerance to autoantigens. Therefore, the autoreactive B cells can be active by self-proteins or molecules and differentiation into plasmablasts or plasma cells, which secrete pathogenic autoantibodies.^[28]^ In anti-LGI1-associated AE, the abnormally secreted anti-LGI1 antibodies disrupt the binding of LGI1 to ADAM22/23, leading to a reduction in postsynaptic AMPA receptors, resulting in excessive network excitability and seizures.^[29]^

Immunomodulatory and immunosuppressive treatments are more efficient in anti-LGI1-associated AE. First-line therapies often include corticosteroids, IVIg and PE. There are potential risks associated with IVIg and PE, and both rely on limited blood resources. Corticosteroids rapidly inhibit inflammation, reduce the destruction of the blood-brain barrier, and as well as improving symptoms in the acute phase. Nevertheless, the prolonged and extensive utilization of corticosteroids has significant side effects, and studies have shown that 27% to 35% of anti-LGI1-associated AE patients treated with corticosteroids alone or tapered too quickly are predisposed to relapse.^[30]^ In this setting, steroid-sparing agents like methotrexate, azathioprine, and mycophenolate mofetil are the more common choice. Nevertheless, relapses still occur. Given the key role of B cells in pathogenesis, the chimeric monoclonal antibody directed against the B-cell marker CD20, rituximab, is widely used in autoimmune encephalitis, and the relapse rate is decreased in cases following rituximab treatment.^[31]^ While adverse reactions such as infections, allergic reactions and leukopenia increased, thus many new targeted drugs were evaluated in clinical trials.

Efgartigimod has been demonstrated to be highly effective and safe in the treatment of myasthenia gravis. Consequently, it is being explored as a potential treatment for other autoimmune diseases, including autoimmune encephalitis. In this case, the patient was initially treated with methylprednisolone pulse therapy, but this treatment failed. We informed the patient’s family about the potential benefits and risks of efgartigimod, after which written informed consent was obtained. The patient was treated with a combination of methylprednisolone and efgartigimod. After 3 weeks of treatment, the patient’s symptoms improved significantly and the abnormal lesions on the cranial MRI scan were attenuated, suggesting that blocking the pathogenic pathway synergistically is more efficient without adverse events or relapses. However, long-term follow-up of patients for recurrence is still required.

Antibodies against LGI1 are predominantly of the IgG4 subtype. IgG4 antibodies do not possess pro-inflammatory properties, rather, they work by inhibing protein-protein interactions with their targets. This reinforces that humoral immunity plays an important role in the pathogenesis of anti-LGI1-associated AE.^[19,32]^ The FcRn controls the trafficking and recycling of the pathogenic IgG. Efgartigimod has a stronger affinity to FcRn compared with endogenous IgG, thereby increasing the degradation of pathogenic IgG.^[8]^ Studies have shown that efgartigimod can reduce IgG levels by 75% and is effective against IgG4 subtypes.^[33]^ Efgartigimod suppresses abnormal autoimmunity by reducing levels of abnormal antibodies. Mitogen-activated protein kinases (MAPK) signaling pathways contribute to the production of pro-inflammatory cytokines such as tumor necrosis factor, interleukin-1β and interleukin-6. These pathways involved in different subdivisions of intracellular inflammatory pathways that modulate immune, inflammatory and neurodegenerative responses.^[34,35]^ Activation of the MAPK signaling pathway promotes the development of autoimmune encephalomyelitis such as multiple sclerosis.^[35]^ Fc receptor for IgG, Fc gamma receptor (FcγR), which is primarily expressed on immune cells such as monocytes, neutrophils, macrophages, and dendritic cells. FcγR generates a well-balanced immune response through triggering of activating and inhibitory signaling pathways. Aberrant expression or the presence of allelic variants of FcγRs with altered functionality has been observed in a variety of human autoimmune diseases, and these contribute to the pathogenesis of these diseases.^[36]^ FcγRs trigger the activation of innate effector cells, and they are also involved in antigen presentation, the immune-complex-mediated maturation of dendritic cells, and the regulation of B-cell activation and plasma cell survival. The MAPK signaling pathway plays a central and crucial role in cell activation following FcγR crosslinking.^[37]^ Research has demonstrated that the expression of Fc receptor γ subunit, a key component of FcγR, was reduced in efgartigimod-treated mice, which dampened FcγR-dependent production of proinflammatory cytokines.^[38]^ Therefore, we speculate that efgartigimod not only reduces levels of pathogenic antibodies, but may also exert therapeutic effects through suppression of FcγR expression and activation, thereby influencing the upstream of MAPK signaling pathway, resulting in a reduction of the pro-inflammatory MAPK cascade. This requires further exploration and verification. The phosphatidylinositol-3-kinase (PI3K) pathway plays a key role in cell growth, differentiation, and metabolism. AKT is a direct downstream effector molecule of the PI3K signaling cascade, while mTOR is a key downstream target of the PI3K/AKT pathway. Furthermore, the PI3K/AKT/mTOR pathway plays an essential role in the process and release of proinflammatory factors.^[39,40]^ Dysfunction of phosphatidylinositol-4,5-bisphosphate 3-kinase catalytic subunit beta can lead to excessive inflammatory responses or immunosuppression.^[41]^ Furthermore, report indicates that the activation of the PI3K/AKT signaling pathway, along with increased mTOR phosphorylation, may play an important role in the progression of experimental autoimmune encephalomyelitis, meanwhile methylprednisolone reduced neuroinflammation in experimental autoimmune encephalomyelitis by inhibition of the PI3K/AKT/mTOR signaling pathways.^[39]^ However, whether efgartigimod regulates the PI3K/AKT/mTOR pathway remains unclear. We will conduct an in-depth exploration into the molecular biological mechanisms of efgartigimod in autoimmune encephalitis.

## 5. Conclusion

In conclusion, the early diagnosis and treatment of autoimmune encephalitis is important. This improves the prognosis of patients. The prevailing immunotherapies have inherent limitations, and some patients exhibits suboptimal responses to contemporary first- and second-line immunotherapies. The present case illustrates that efgartigimod rapidly and effectively ameliorates the symptoms and imaging abnormalities of anti-LGI1-associated AE. This suggests that efgartigimod may be a viable treatment option for anti-LGI1-associated AE. Currently, there are few case reports of efgartigimod in the treatment of autoimmune encephalitis. Thus, longitudinal, multicenter studies are needed to determine the optimal frequency and dosage of efgartigimod treatment, as well as to clarify the long-term safety and efficacy. Meanwhile, the molecular biological mechanisms by which efgartigimod modulates immunity require further exploration.

## Author contributions

**Conceptualization:** Rui Gao, Yan Hong, Guanen Zhou.

**Writing – original draft:** Rui Gao.

**Writing – review & editing:** Yan Hong, Guanen Zhou.
